# Exposure to E-Cigarette Advertisements or Reviews and E-Cigarette Use Progression: A Longitudinal Examination of Short-Term and Long-Term Associations among US Young Adults

**DOI:** 10.3390/ijerph21020123

**Published:** 2024-01-23

**Authors:** Zongshuan Duan, Katelyn F. Romm, Yan Wang, Jidong Huang, Carla J. Berg

**Affiliations:** 1 Department of Population Health Sciences, School of Public Health, Georgia State University, Atlanta, GA 30302, USA; 2 Department of Pediatrics, College of Medicine, University of Oklahoma Health Sciences Center, Oklahoma City, OK 73126, USA; katelyn-romm@ouhsc.edu; 3 Department of Prevention and Community Health, Milken Institute School of Public Health, George Washington University, Washington, DC 20052, USA; yanwang20@email.gwu.edu (Y.W.); carlaberg@email.gwu.edu (C.J.B.); 4 Department of Health Policy & Behavioral Sciences, School of Public Health, Georgia State University, Atlanta, GA 30302, USA; jhuang17@gsu.edu; 5 George Washington Cancer Center, George Washington University, Washington, DC 20052, USA

**Keywords:** e-cigarettes, advertisement, review, use pattern, progression

## Abstract

Limited research has investigated the impact of e-cigarette advertising and reviews on the progression of e-cigarette use among young adults in the US. This study utilized five-wave longitudinal data (2018–2020) with 3006 young adults aged 18–34, reporting exposure to e-cigarette advertisements or reviews at Wave 1 (W1) and W3. Generalized estimating equations (GEE) were used to examine the prospective associations between frequent exposure to e-cigarette advertisements or reviews and e-cigarette use progression in four groups: never users (n = 1271 at W1), former users (previously used but quit ≥ 6 months ago, n = 422 at W1), recent former users (used in the past 6 months but not in the past month, n = 186 at W1), and current users (used in the past month, n = 1127 at W1). Among baseline former users, frequent exposure to e-cigarette reviews was associated with current use at 6-month follow-up (aOR = 4.40, 95%CI = 1.46–13.29). Among baseline current users, frequent exposure to e-cigarette reviews was associated with increased days of use at 6-month follow-up (IRR = 1.20, 95%CI = 1.07–1.34) and 12-month follow-up (IRR = 1.18, 95%CI = 1.03–1.35). E-cigarette reviews may contribute to relapse among recent former users and increased usage frequency among current users, highlighting the need for enhanced e-cigarette promotional activity regulation.

## 1. Introduction

Since their introduction to the United States market in 2007, e-cigarettes have experienced a significant increase in popularity, particularly among young adults [1]. The prevalence of current (past 30-day) e-cigarette use among young adults aged 18–24 in the US rose from 5.1% in 2014 to 11.0% in 2021 [2,3]. This period also witnessed a substantial growth in the demand for e-cigarettes. Retail scanner data from Information Resources, Inc. (IRI) indicated a nearly 300% increase in total e-cigarette unit sales in the US between 2016 and 2019 [4]. However, sales of e-cigarettes experienced a decline during the period of August 2019 to February 2020, which may have been partially influenced by the outbreak of e-cigarette, or vaping, product use-associated lung injury (EVALI) [4]. Despite federal and state legalization raising the legal tobacco purchase age to 21 and the enforcement of restrictions on flavored e-cigarette products [5], e-cigarette sales still saw a 46.3% increase from February 2020 to March 2021 [6].

Existing literature suggests that the growing popularity of e-cigarette use can be attributed, at least in part, to increased awareness and positive perceptions of e-cigarette products, influenced by e-cigarette marketing [7,8,9,10]. Unlike combustible cigarettes, which have been banned from television and radio advertising since 1971, e-cigarette advertising remained largely unregulated in the US until recently [1]. The primary sources of e-cigarette advertising exposure included retail stores, the internet, television, and print media [11]. Early e-cigarette advertising mainly took place on television and in print media, employing strategies borrowed from conventional cigarette marketing. These advertisements often positioned e-cigarette products as a healthier alternative to combustible cigarettes, showcasing a fashionable lifestyle [12]. They frequently targeted young people by featuring celebrities, cultural icons, models, and athletes, while highlighting sleek product designs and a variety of flavors [1,10]. In recent years, e-cigarette products have been aggressively promoted on social media platforms such as YouTube, Instagram, Facebook, and Twitter through official company accounts, affiliated accounts, and paid influencers [1].

Previous studies, including both observational studies and randomized controlled trials, have consistently shown that exposure to e-cigarette advertising is significantly associated with e-cigarette use and susceptibility among US adolescents [10,13,14,15,16,17]. However, there is limited empirical evidence regarding the prospective relationship between e-cigarette advertising exposure and e-cigarette use among US young adults. Only a few published studies have examined the association between e-cigarette advertising exposure and e-cigarette use among US adults. An analysis of 2013-14 National Adult Tobacco Survey (NATS) data revealed that exposure to e-cigarette advertisements on TV was associated with increased awareness, ever use, and current use of e-cigarettes among adults [18]. Furthermore, a web-based experimental study found that US adults exposed to e-cigarette ads were more likely to express interest in trying e-cigarettes [19]. Additionally, Nicksic et al., (2017) documented a positive association between exposure to tobacco advertising outlets and e-cigarette use and susceptibility [20]. However, most of these previous studies relied primarily on cross-sectional data, emphasizing the need for longitudinal studies to better understand the relationship between e-cigarette advertising and use among US adults, particularly young adults who exhibit a significantly higher prevalence of e-cigarette use compared to other age groups of adults [21,22].

Moreover, previous studies have predominantly focused on the exposure to paid e-cigarette advertisements [10,13,14,15,16,17], overlooking the potential impact of e-cigarette reviews or recommendations on e-cigarette use (e.g., promotion content on television, radio, websites, and social media) [23,24]. However, findings from some studies have suggested that product reviews or recommendations can effectively influence e-cigarette use among young individuals [25,26,27]. Studies exploring the role of social media micro-influencers have revealed that young adults perceive e-cigarette-related posts made by sponsored users as more trustworthy compared to posts from official brand accounts [4]. Additionally, content shared by social media influencers is often considered more reliable and authentic than traditional advertising methods [25]. Previous reports have also indicated close collaborations between influencers and e-cigarette companies, with influencers posting e-cigarette-related reviews or recommendations through their channels [25,27]. Consequently, it is crucial to comprehend the associations between exposure to e-cigarette reviews or recommendations and e-cigarette use behaviors to guide potential regulatory policies and develop effective public health communication strategies.

Furthermore, previous studies primarily focused on the association between exposure to e-cigarette advertising and e-cigarette initiation, but not relapse or changes in frequency of use. Drawing from the evidence on the effects of cigarette marketing, exposure to cigarette advertising/promotion is significantly associated with the progression of cigarette smoking states (e.g., initiation among never users, increasing use frequency among current users) [28,29]. However, there is limited empirical evidence about the longitudinal relationship between e-cigarette marketing and e-cigarette use progression among US young adults.

The primary objective of this study is to address these significant research gaps by utilizing data from a large and diverse cohort of young adults in the United States, aged 18–34 years, across six metropolitan statistical areas (MSAs) from 2018 to 2020. We aim to explore the prospective associations between exposure to e-cigarette advertisements and reviews, and the progression of e-cigarette use behaviors within specific subgroups, including baseline nonusers, former users, and current users. We hypothesized that, among US young adults, exposure to e-cigarette advertisements or reviews were prospectively associated with: (1) initiating e-cigarette use among never users, (2) becoming current users among former users, and (3) increasing number of days using e-cigarettes among current users.

## 2. Materials and Methods

### 2.1. Study Design and Participants

This study analyzed Wave 1 (W1) to W5 survey data from 3006 young adults (18–34 years old) who participated in the Vape shop Advertising, Place characteristics and Effects Surveillance (VAPES) study. The VAPES study is a 2-year, 5-wave cohort study, examining the e-cigarette retail environment and its impacts on tobacco use. This study was approved by the Emory University Institutional Review Board. The detailed study design and data collection procedures are published elsewhere [30] and summarized here. Starting in September 2018, the VAPES study recruited participants from 6 MSAs: Atlanta, Georgia; Boston, Massachusetts; Minneapolis, Minnesota; Oklahoma City, Oklahoma; San Diego, California; and Seattle, Washington. These MSAs were selected for their variations in state tobacco control policies [31]. Potential participants were recruited via social media. Eligibility criteria were: (1) 18–34 years old; (2) living in one of the 6 MSAs, as indicated by residential zip code; and (3) English speaking. Purposive, quota-based sampling was used to ensure sufficient representation of e-cigarette and cigarette users (~1/3 each), sexes, and racial/ethnic minorities. Participants who completed enrollment were immediately administered the W1 survey. In the final baseline sample (n = 3006; 42.3% male, 71.6% White, 11.4% Hispanic), 26.9% (n = 808) reported past 30-day cigarette use and 37.7% (n = 1133) reported past 30-day e-cigarette use. Considering overlap in use groups, 19.0% (n = 571) reported dual cigarette/e-cigarette use, 7.9% (n = 237) exclusive cigarette use, 18.7% (n = 562) exclusive e-cigarette use, and 54.4% (n = 1636) no use of cigarettes or e-cigarettes. Subsequent assessments were conducted every 6 months (Fall 2018 to Fall 2020), totaling 5 waves (retention rates: W2 [Spring 2019]: n = 2551, 84.9%; W3 [Fall 2019]: n = 2375, 79.0%; W4 [Spring 2020]: n = 2159, 71.8%; W5 [Fall 2020]: n = 2476, 82.4%).

The current study conducted analyses using data collected from all five waves to investigate both short-term and long-term associations between exposure to e-cigarette advertisements and reviews, and changes in e-cigarette use behaviors. Specifically, we examined the short-term (6-month) associations between e-cigarette advertising and review exposure at baseline (W1 and W3) and subsequent changes in e-cigarette use status at follow-up (W1 to W2, and W3 to W4, respectively). Furthermore, we explored the long-term (12-month) associations between baseline exposure to e-cigarette advertisements and reviews and changes in e-cigarette use status (W1 to W3, and W3 to W5, respectively). The sample sizes and distribution of e-cigarette use status at each wave are visually presented in Supplementary Figure S1.

This study was approved by the institutional review boards of Emory University Institutional Review Board (IRB00097895), and informed consent was obtained from all participants. The study results are presented according to the Strengthening the Reporting of Observational Studies in Epidemiology (STROBE) guideline for cohort studies.

### 2.2. Measures

#### 2.2.1. Primary Outcomes: Change in E-Cigarette Use Behaviors

To investigate changes in e-cigarette use behaviors, we first created e-cigarette use categories, using participants’ responses to 3 questions assessing lifetime use at W1 (yes/no), and number of days of use in the past 6 months and past 30 days, respectively, at each wave. At W1, never users were participants who reported no lifetime use. At follow-up waves, never users were those who reported no lifetime use at W1 and no use of e-cigarettes in the past 6 months at the respective wave of data collection and all prior follow-up waves. Baseline never e-cigarette users who reported that they ever used any e-cigarette at follow-up were classified as initiated e-cigarette use for the wave in which initiation occurred.

Former users were those who reported lifetime e-cigarette use but no use in the past 6 months. Recent former users were those who reported past 6-month use but no past 30-day use. Current users were those who reported any past 30-day e-cigarette use. Among current users, use frequency was operationalized as the number of days of use in the past 30 days.

#### 2.2.2. Primary Predictors: Exposure to E-Cigarette Advertisements or Reviews at W1 and W3

In W1 and W3 assessments, participants were asked “During the past 30 days, how many times you have you seen *ads/promotions, coupons, or discounts* for a vape shop, buying an e-cigarette device or vaporizer, or buying e-liquids or e-juices?” and “During the past 30 days, how many times you have you seen *reviews or recommendations* for vape shops, e-cigarette devices or vaporizers, or e-liquids or e-juices?” Responses of “3–5 times”, “6–10 times”, or “More than 10 times” were classified as frequent exposure; responses of “Not in the past 30 days” or “1–2 times” were classified as infrequent exposure.

#### 2.2.3. Covariates: Sociodemographic Characteristics and Other Tobacco Use at W1 and W3

We included MSA (Atlanta, Boston, Minneapolis, Oklahoma City, San Diego, Seattle), age, gender (male, female, other), race/ethnicity (non-Hispanic white, non-Hispanic black, non-Hispanic other, Hispanic), education level (<college, ≥college), employment (employed, unemployed), sexual orientation (minority, heterosexual), marital status (married/cohabitating, other), having child (yes/no), past 6-month cigarette use (yes/no), and past 6-month other tobacco product use (little cigars/cigarillos, hookah, and smokeless tobacco; yes/no) as covariates in the analyses.

### 2.3. Data Analysis

All analyses were conducted separately based on participants’ baseline (W1 and W3) e-cigarette use status (never users, former users, recent former users, and current users). Descriptive analyses were used to summarize outcomes, predictors, and covariates. Bivariate analyses (Chi-Square tests for categorical variables; 2-sample *t*-tests and analysis of variance (ANOVA) for continuous variables) were used to characterize crude differences. Generalized estimating equations (GEE) models were used to estimate the adjusted associations. Specifically, we examined the prospective short-term (6-month) and long-term (12-month) associations between exposure to e-cigarette advertisements or reviews and 4 categories of e-cigarette use progression behaviors: (1) initiating e-cigarette use at follow-up among baseline never users, (2) becoming past 6-month users at follow-up among baseline former users, (3) becoming current users at follow-up among baseline recent former users, and (4) becoming more frequent e-cigarette users at follow-up among baseline current users. Sensitivity analyses using exposure to e-cigarette advertisements or reviews as continuous variables at Wave 1 and Wave 3 were conducted to test the robustness of these associations. Stata 15.1 (Stata Corp LLC: College Station, TX, USA) was used for analyses.

## 3. Results

### 3.1. Participant Characteristics

Table 1 presents the proportions of frequent exposure to e-cigarette ads or reviews, and the socio-demographic characteristics of the study sample by e-cigarette use status at baseline waves (W1 and W3). At W1, frequent exposure to e-cigarette advertisements and reviews, respectively, was reported by 31.9% and 10.0% of never users, 28.9% and 8.3% of former users, 33.3% and 13.4% of recent former users, and 37.4% and 29.6% of current users. At W3, frequent exposure to e-cigarette advertisements and reviews, respectively, was reported by 40.5% and 11.5% of never users, 43.4% and 16.7% of former users, 35.4% and 13.3% of recent former users, and 39.3% and 22.3% of current users. Detailed descriptive statistics for other covariates at Wave 1 and Wave 3 by e-cigarette use status are available in Supplementary Table S1.

We also conducted bivariate analyses to explore covariates in relation to e-cigarette ads and review exposure (Supplementary Table S2). E-cigarette ad exposure at W1 was significantly associated with frequent exposure to e-cigarette reviews, MSA, race, employment status, marital status, having child, past 6-month cigarette and other tobacco use. In addition, e-cigarette review exposure at W1 was significantly associated with all covariates except for sexual minority status. At W3, exposure to e-cigarette ads was associated with exposure to e-cigarette reviews, MSA, race, marital status, and past 6-month other tobacco use; exposure to e-cigarette reviews was associated with all covariates except for gender and sexual minority status.

### 3.2. Associations between E-Cigarette Ads or Reviews and Changes in E-Cigarette Use

Table 2 presents the percentages of e-cigarette use progressions at 6-month and 12-month follow-ups by baseline e-cigarette use status and exposure to e-cigarette ads or reviews. E-cigarette initiation at W2 among W1 never users was significantly higher among those who reported frequent exposure to e-cigarette reviews (15.1% vs. 7.1%; *p* = 0.007). W1 recent former users were more likely to become current users at W2 among those who report frequent exposure to e-cigarette reviews (57.1% vs. 29.9%; *p* = 0.023). W3 former users were more likely to become past 6-month users at W4 among those who reported frequent exposure to e-cigarette reviews (22.7% vs. 12.8%; *p* = 0.043). In addition, during a 12-month follow-up period, W1 never users were significantly more likely to initiate e-cigarette use at W3 among those who reported frequent exposure to e-cigarette reviews (15.3% vs. 8.7%; *p* = 0.043). No significant associations between e-cigarette ad exposure and changes in e-cigarette use were identified.

Table 3 shows the adjusted associations between frequent exposure to e-cigarette advertisements or reviews and progressions of e-cigarette use behaviors at short-term (6-month) follow-up waves, controlling for MSAs and other individual characteristics, by baseline e-cigarette use status. Among baseline recent former users, frequent exposure to e-cigarette *reviews* at baseline was associated with increased odds of current use at 6-month follow-up (aOR = 4.40, 95% CI: 1.46–13.29). Among baseline current users, exposure to e-cigarette *ads* at baseline was associated with reduced number of days using e-cigarettes at 6-month follow-up (IRR = 0.88, 95% CI: 0.79–0.99); yet, frequent exposure to e-cigarette *reviews* predicted increased number of days using e-cigarettes at 6-month follow-up (IRR = 1.20, 95% CI: 1.07–1.34).

Table 4 shows the adjusted associations between frequent exposure to e-cigarette advertisements or reviews and progressions of e-cigarette use behaviors at long-term (12-month) follow-up waves. Among baseline former users, frequent exposure to e-cigarette ads at baseline was associated with reduced odds of past 6-month use at 12-month follow-up (aOR = 0.65, 95% CI: 0.43–0.98). Among baseline current users, frequent exposure to e-cigarette reviews predicted an increased number of days using e-cigarettes at 12-month follow-up (IRR = 1.18, 95% CI: 1.03–1.35).

To examine the robustness of our findings, we conducted sensitivity analyses by replacing categorical exposure to e-cigarette ads or reviews with continuous measures. The results are presented in Supplementary Table S3, which shows similar results regarding the associations between exposure to e-cigarette ads or reviews and e-cigarette use trajectories. Specifically, at 6-month follow-up, exposure to e-cigarette reviews at baseline was associated with increased odds of becoming current users among baseline recent former users (aOR = 1.76, 95% CI: 1.13–2.73), and increased number of days using e-cigarettes among baseline current users (IRR = 1.09, 95% CI: 1.04–1.14). In the 12-month follow-up, exposure to e-cigarette reviews at baseline was associated with the increased number of days using e-cigarettes among baseline current users (IRR = 1.07, 95% CI: 1.01–1.13).

## 4. Discussion

This study prospectively examined the exposure to e-cigarette ads and reviews in relation to e-cigarette use progression among US young adults. Notably, we distinguished exposure to e-cigarette ads versus reviews for e-cigarettes, which proved to be a critical distinction. Among baseline former users, frequent exposure to e-cigarette reviews was associated with a greater likelihood of becoming current users at the 6-month follow-up. In addition, among baseline current users, frequent exposure to e-cigarette reviews was prospectively associated with an increased number of days using e-cigarettes at 6-month and 12-month follow-ups. However, frequent exposure to e-cigarette ads did not predict e-cigarette use progression among any subgroup, and in the short-term, predicted fewer days using e-cigarettes among current users.

Current findings that e-cigarette advertising did not predict use progression appear contradictory to previous studies, which indicate that exposure to e-cigarette advertisements may be associated with increased interest [19], susceptibilities [20], and ever and current use of e-cigarettes [18]. The discrepancies in findings may be due to the fact that previous studies mainly utilized cross-sectional data and did not examine changes in e-cigarette use among subgroups characterized by e-cigarette use status. In addition, most previous studies failed to distinguish e-cigarette ads from reviews or recommendations. Therefore, respondents may perceive reviews or recommendations as a particular type of e-cigarette advertising. Qualitatively, e-cigarette advertisements and reviews may differ in both presentation and intent. E-cigarette advertising aims to promote the product by utilizing visual appeals and persuasive messages [32,33], while e-cigarette reviews or recommendations may focus more on reflecting individual experiences and opinions, offering nuanced insights [25]. Recognizing these distinctions is crucial for future studies to examine the impact of e-cigarette promotional activities on user progression. Nonetheless, given the correlations between e-cigarette marketing and awareness and perceptions [7,8,9,10], it is important to place stricter evidentiary standards on e-cigarette promotional activities, including advertisements and reviews.

Consistent with our hypotheses, results showed the associations between frequent exposure to e-cigarette reviews and e-cigarette use progression, including becoming current users at 6-month follow-up among baseline recent former users, and increased number of days using e-cigarettes at 6-month and 12-month follow-ups among baseline current users. This coincides with prior findings that exposure to social media advertising featuring e-cigarettes is associated with lower perceived risks, stronger use intentions [25,34,35,36], and increased use behaviors [36,37,38,39], and with the increasing evidence regarding influencers’ role in tobacco promotion [27,40,41]. FDA has engaged in several regulatory actions to reduce advertising exposure among young people, including requesting e-cigarette companies to provide practices regarding social media influencer promotion and managing followers to restrict young people’s exposure and appeal [42]. However, this content is still present on major social media platforms and seen among both e-cigarette users and nonusers. In the current study, approximately 10% of never e-cigarette users were exposed to such marketing.

Aligning with previous longitudinal studies [43,44,45], baseline cigarette and other tobacco use were found to be positively associated with e-cigarette use trajectories. Furthermore, consistent with the overall prevalence of e-cigarette use [22,43,46], respondents who were race or ethnic minorities were less associated with e-cigarette use progression. In addition, participants from cities with more strict state policies regarding tobacco control and the legalization status of recreational cannabis were associated with an increased likelihood of e-cigarette use progression. Residents in MSAs with more strict tobacco control regulatory efforts (e.g., Sand Diego and Boston, followed by Seattle, Minneapolis, and Oklahoma City in comparison to Atlanta based on the STATE system of the US CDC [47]) may have a stronger incentive to use e-cigarettes as an alternative to regular cigarettes, particularly in places where traditional cigarettes use is prohibited [48]. More comprehensive tobacco control regulations, including e-cigarette-inclusive smoke-free air policy and a higher e-cigarette excise tax, may be needed to effectively reduce young adult e-cigarette use [49,50,51].

The current research findings have important implications for both research and policy. Given the distinct impacts of exposure to advertising and reviews, these findings emphasize the need to distinguish these factors and provide the basis for future research exploring the overlap and interactive effects of exposure to advertising and reviews with regard to individuals’ tobacco use behaviors over time [52]. Furthermore, these findings suggest that diversified marketing strategies, such as utilizing reviews or recommendations, may become increasingly important for e-cigarette companies targeting young adults. To address this issue, regulatory action is necessary to prohibit sponsored e-cigarette content on social media platforms, particularly reviews and recommendations from influencers who appeal to young people. The US FDA has taken some regulatory measures, such as requesting e-cigarette companies to disclose their practices regarding social media influencer promotion and follower management to restrict young people’s exposure and appeal [42]. However, sponsored e-cigarette content can still be found on major social media channels [23,24], and no regulatory actions have been implemented to restrict adult access to e-cigarette marketing on any media platforms in the US [53]. Efforts to reduce adult e-cigarette use should consider implementing measures that limit adult access to e-cigarette marketing across various media channels. This is particularly important because the study found high rates of exposure to e-cigarette advertisements and reviews among adults, regardless of their e-cigarette use status.

However, this study has several limitations. Firstly, all the measures used in this study were self-reported, which may introduce recall bias [54]. Secondly, the study relied on a nonprobability sample that oversampled cigarette and e-cigarette users, so caution should be exercised when generalizing the findings to the population level or other regions in the US. Thirdly, although temporal associations were observed, the study design could not establish causal relationships between e-cigarette ads or reviews and e-cigarette use behaviors. Fourthly, a small size for the subsample may prevent the findings from being extrapolated and may introduce spurious effects [55]. Additionally, measures used in this study did not assess where reviews were seen or differentiate sponsored and non-sponsored reviews; thus, future studies may consider distinguishing exposure to reviews circulated via different channels (e.g., social media, online retail websites) and with regard to source of the reviews (e.g., industry-sponsored). Furthermore, our measures did not explicitly state that the intent was to assess exposure to e-cigarette promotions (rather than anti-tobacco content) in the advertising or reviews. As such, ensuring that this is clarified in future measures is important, and future studies may also explore anti-e-cigarette content exposure and its interactive effects with promotional content exposure. Finally, despite controlling for various covariates, the models did not account for other potentially important confounding factors.

## 5. Conclusions

Collectively, the current findings highlight the importance of specificity in assessments of e-cigarette advertising exposure versus exposure to reviews and recommendations and of examining the impact among subgroups of young adults based on their e-cigarette use status. While exposure to e-cigarette ads did not predict e-cigarette use progression, exposure to e-cigarette reviews or recommendations predicted progression among recent former users and current users, thereby potentially encouraging e-cigarette use behaviors. Continuous monitoring of the type and channel of e-cigarette advertising exposure, as well as its impact on e-cigarette use among young adults, is essential. Policies comprehensively regulating the marketing of e-cigarette products among young adults are critical, particularly measures aimed at effectively reducing the availability of e-cigarette reviews.

## Figures and Tables

**Table 1:** Baseline exposure to e-cigarette ads or reviews in the past 30 days and demographics by baseline e-cigarette use status.

	Never Users	Former Users	Recent Users	Current Users	
	N = 1271	N = 422	N = 186	N = 1127	
	% (n)	% (n)	% (n)	% (n)	*p*
Wave 1					
Exposure to e-cigarette ads					
Frequent	31.9 (405)	28.9 (122)	33.3 (62)	37.4 (422)	0.004
Not frequent	68.1 (866)	71.1 (300)	66.7 (124)	62.6 (705)	
Exposure to e-cigarette reviews					
Frequent	10.0 (127)	8.3 (35)	13.4 (25)	29.6 (333)	<0.001
Not frequent	90.0 (1144)	91.2 (387)	86.6 (161)	70.5 (794)	
Wave 3					
Exposure to e-cigarette ads					
Frequent	40.5 (370)	43.4 (203)	35.4 (56)	39.3 (305)	0.293
Not frequent	59.5 (543)	56.6 (265)	64.6 (102)	60.7 (471)	
Exposure to e-cigarette reviews					
Frequent	11.5 (105)	16.7 (78)	13.3 (21)	22.3 (173)	<0.001
Not frequent	88.5 (808)	83.3 (390)	86.7 (137)	77.7 (603)	

Note: Never users were participants who reported no lifetime use; Former users were those who reported lifetime e-cigarette use but no use in the past 6 months; Recent users were those who reported past 6-month use but no past 30-day use; Current users were those who reported any past 30-day e-cigarette use.

**Table 2:** Prevalence of e-cigarette use at follow-up waves by baseline e-cigarette use status and exposure to e-cigarette ads or reviews in the past 30 days.

	Exposure to E-Cigarette Ads		Exposure to E-Cigarette Reviews	
	Frequent % (n)	Not Frequent % (n)	Frequent % (n)	Not Frequent % (n)
Short-term follow-up W1–W2				
Initiation among never user	9.1 (33)	7.2 (56)	15.1 (16)	7.1 (73)
No	90.9 (328)	92.8 (720)	84.9 (90)	92.9 (958)
Former user become past 6-month user	25.0 (28)	29.0 (74)	32.3 (10)	27.4 (92)
No	75.0 (84)	71.0 (181)	67.7 (21)	72.6 (244)
Recent former users become current user	39.6 (21)	30.4 (34)	57.1 (12)	29.9 (43)
No	60.4 (32)	69.6 (78)	42.9 (9)	70.1 (101)
Use frequency, M (SD)	15.42 (12.78)	15.57 (13.06)	16.38 (12.71)	15.14 (13.04)
Short-term follow-up W3–W4				
Initiation among never user	2.6 (8)	3.3 (15)	1.2 (1)	3.2 (22)
No	97.4 (300)	96.7 (442)	98.8 (80)	96.8 (662)
Former user become past 6-month user	12.5 (21)	15.9 (37)	22.7 (15)	12.8 (43)
No	87.5 (147)	84.1 (196)	77.3 (51)	87.2 (292)
Recent former users become current user	18.2 (8)	14.3 (12)	29.4 (5)	13.5 (15)
No	81.8 (36)	85.7 (72)	70.6 (12)	86.5 (96)
Use frequency, M (SD)	12.22 (12.88)	12.29 (13.11)	13.86 (12.67)	11.84 (13.08)
Long-term follow-up W1–W3				
Initiation among never user	9.2 (31)	9.3 (70)	15.3 (15)	8.7 (86)
No	90.8 (307)	90.7 (680)	84.7 (83)	91.3 (904)
Former user become past 6-month user	25.7 (27)	32.6 (78)	43.3 (13)	29.3 (92)
No	74.3 (78)	67.4 (161)	56.7 (17)	70.7 (222)
Recent former users become current user	28.3 (13)	27.6 (29)	42.1 (8)	25.8 (34)
No	71.7 (33)	72.4 (76)	57.9 (11)	74.2 (98)
Use frequency, M (SD)	13.4 (12.56)	12.7 (12.98)	11.58 (12.95)	11.24 (13.11)
Long-term follow-up W3–W5				
Initiation among never user	1.7 (6)	2.1 (11)	0 (0)	2.2 (17)
No	98.3 (352)	97.9 (506)	100 (101)	97.8 (757)
Former user become past 6-month user	13.0 (25)	17.1 (44)	17.8 (13)	14.9 (56)
No	87.0 (167)	82.9 (213)	82.2 (60)	85.1 (320)
Recent former users become current user	28.6 (14)	19.8 (19)	31.6 (6)	21.4 (27)
No	71.4 (35)	80.2 (77)	68.4 (13)	78.6 (99)
Use frequency, M (SD)	13.8 (12.45)	12.61 (12.97)	13.22 (13.42)	10.85 (12.89)

Note: Significant Chi-Square/*t*-tests for bivariate associations (*p* < 0.05) for short- (W1–W2) and long-term (W1–W3) associations between exposure to e-cigarette reviews and e-cigarette initiation among baseline never users; short-term (W1–W2) association between exposure to e-cigarette reviews and becoming current users among baseline recent former users; and short-term (W3–W4) association between exposure to e-cigarette reviews and becoming past 6-month users among baseline former users. The abbreviation M (SD) represents the mean (standard deviation).

**Table 3:** Adjusted associations between exposure to e-cigarette ads or reviews in the past 30 days and e-cigarette use at short-term (6-month) follow-up waves by baseline e-cigarette use status.

Baseline Sample	Never Users	Former Users	Recent Former Users	Current Users
E-Cigarette Use at Short-Term (6-Month) Follow-Up Waves	Initiated Use	Reported P6M Use	Reported Current Use	Number of Days Using
	aOR (95% CI)	aOR (95% CI)	aOR (95% CI)	IRR (95% CI)
Exposure to e-cigarette ads				
Frequent	1.06 (0.65–1.73)	0.67 (0.42–1.08)	0.70 (0.32–1.53)	**0.88 (0.79–0.99)**
Not frequent	Ref	Ref	Ref	**Ref**
Exposure to e-cigarette reviews				
Frequent	0.96 (0.44–2.09)	1.33 (0.73–2.44)	**4.40 (1.46–13.29)**	**1.20 (1.07–1.34)**
Not frequent	Ref	Ref	**Ref**	**Ref**
Age	0.95 (0.90–1.00)	**0.93 (0.87–0.98)**	0.89 (0.80–0.99)	1.01 (1.00–1.02)
MSA				
Atlanta	Ref	**Ref**	Ref	Ref
Boston	1.02 (0.53–1.96)	**3.27 (1.51–7.08)**	2.45 (0.84–7.16)	**0.82 (0.69–0.98)**
Minneapolis	0.76 (0.34–1.72)	**3.23 (1.46–7.12)**	1.75 (0.58–5.25)	**1.23 (1.06–1.43)**
Oklahoma City	0.83 (0.34–2.02)	**4.84 (1.89–12.35)**	1.46 (0.28–7.59)	1.08 (0.90–1.31)
San Diego	1.27 (0.63–2.54)	**3.66 (1.60–8.38)**	3.04 (0.90–10.30)	1.03 (0.87–1.22)
Seattle	0.65 (0.26–1.64)	**2.78 (1.16–6.67)**	1.07 (0.29–3.92)	1.05 (0.89–1.24)
Other		1.01 (0.16–6.27)		0.64 (0.35–1.18)
Sex				
Male	1.00 (0.63–1.58)	0.74 (0.49–1.10)	0.87 (0.44–1.72)	0.93 (0.84–1.03)
Female	0.86 (0.17–4.37)	1.15 (0.42–3.15)	1.21 (0.17–8.91)	1.01 (0.74–1.39)
Other	Ref	Ref	Ref	Ref
Race				
Non-Hispanic White	Ref	Ref	Ref	Ref
Non-Hispanic Black	0.95 (0.34–2.69)	1.00 (0.36–2.76)	**8.50 (1.87–38.68)**	**0.37 (0.24–0.57)**
Non-Hispanic Other	1.40 (0.79–2.47)	1.12 (0.65–1.93)	1.25 (0.50–3.11)	**0.83 (0.73–0.96)**
Hispanic	1.48 (0.69–3.15)	0.58 (0.29–1.15)	0.70 (0.22–2.27)	**0.79 (0.68–0.92)**
Education (Ref: <college)	0.82 (0.46–1.46)	1.18 (0.68–2.06)	0.95 (0.41–2.18)	**0.84 (0.76–0.92)**
Employment (Ref: unemployed)	1.52 (0.90–2.57)	1.10 (0.68–1.78)	0.60 (0.30–1.20)	1.06 (0.95–1.18)
Sexual minority (Ref: No)	**1.99 (1.21–3.27)**	1.24 (0.77–2.00)	0.60 (0.31–1.14)	1.02 (0.92–1.14)
Married (Ref: No)	0.78 (0.45–1.35)	0.76 (0.48–1.21)	1.14 (0.46–2.86)	**1.16 (1.05–1.29)**
Having child (Ref: No)	0.87 (0.35–2.17)	1.78 (0.97–3.27)	2.15 (0.63–7.35)	0.98 (0.85–1.13)
Other tobacco use, past 6 months				
Cigarettes (Ref: No)	**11.38 (6.41–20.20)**	**4.61 (2.92–7.28)**	**3.74 (1.77–7.90)**	1.06 (0.96–1.16)
Other tobacco products (Ref: No) ^	**3.70 (2.07–6.61)**	**3.90 (2.44–6.23)**	1.69 (0.75–3.79)	1.02 (0.93–1.13)

^ Other tobacco included little cigars/cigarillos, hookah, and smokeless tobacco; boldface indicates *p* < 0.05. Never users were participants who reported no lifetime use; Former users were those who reported lifetime e-cigarette use but no use in the past 6 months; Recent users were those who reported past 6-month use but no past 30-day use; Current users were those who reported any past 30-day e-cigarette use. The term “Ref” denotes reference.

**Table 4:** Adjusted associations between exposure to e-cigarette ads or reviews in the past 30 days and e-cigarette use at long-term (12-month) follow-up waves by baseline e-cigarette use status.

Baseline Sample	Never Users	Former Users	Recent Former Users	Current Users
E-Cigarette Use at Short-Term (12-Month) Follow-Up Waves	Initiated Use	Reported P6M Use	Reported Current Use	Number of Days Using
	aOR (95% CI)	aOR (95% CI)	aOR (95% CI)	IRR (95% CI)
Exposure to e-cigarette ads				
Frequent	0.73 (0.42–1.25)	**0.65 (0.43–0.98)**	0.90 (0.40–2.01)	0.95 (0.83–1.08)
Not frequent	Ref	**Ref**	Ref	Ref
Exposure to e-cigarette reviews				
Frequent	1.24 (0.57–2.67)	1.42 (0.82–2.45)	1.70 (0.56–5.18)	**1.18 (1.03–1.35)**
Not frequent	Ref	Ref	Ref	**Ref**
Age	**0.91 (0.86–0.97)**	**0.91 (0.85–0.96)**	0.98 (0.89–1.08)	1.01 (0.99–1.02)
MSA				
Atlanta	Ref	Ref	Ref	Ref
Boston	0.86 (0.46–1.59)	1.85 (0.95–3.57)	1.96 (0.66–5.77)	**0.68 (0.55–0.82)**
Minneapolis	0.48 (0.20–1.13)	0.98 (0.48–2.01)	**3.57 (1.14–11.24)**	1.01 (0.86–1.19)
Oklahoma City	0.71 (0.29–1.74)	**2.82 (1.32–6.03)**	1.77 (0.37–8.61)	0.89 (0.72–1.09)
San Diego	1.23 (0.64–2.34)	1.50 (0.75–3.02)	3.13 (0.89–10.97)	0.92 (0.77–1.09)
Seattle	1.20 (0.56–2.54)	1.30 (0.62–2.73)	1.65 (0.38–7.11)	0.89 (0.75–1.07)
Other	0.41 (0.03–4.81)	0.53 (0.10–2.91)	**13.26 (1.03–170.68)**	0.72 (0.42–1.24)
Sex				
Male	0.92 (0.60–1.42)	0.94 (0.61–1.45)	0.97 (0.50–1.87)	**0.89 (0.79–1.00)**
Female	0.52 (0.09–2.91)	0.75 (0.24–2.38)		0.97 (0.67–1.41)
Other	Ref	Ref	Ref	Ref
Race				
Non-Hispanic White	Ref	Ref	Ref	Ref
Non-Hispanic Black	0.70 (0.25–1.94)	0.76 (0.30–1.93)	2.02 (0.28–14.63)	**0.37 (0.26–0.53)**
Non-Hispanic Other	1.26 (0.76–2.08)	0.97 (0.56–1.67)	1.58 (0.66–3.82)	**0.79 (0.67–0.93)**
Hispanic	0.91 (0.42–1.94)	0.78 (0.42–1.45)	0.82 (0.20–3.42)	**0.74 (0.62–0.88)**
Education (Ref: <college)	0.59 (0.34–1.01)	0.87 (0.53–1.42)	0.71 (0.31–1.61)	**0.84 (0.75–0.95)**
Employment (Ref: unemployed)	**1.85 (1.08–3.17)**	0.84 (0.54–1.31)	0.95 (0.46–1.96)	**1.17 (1.03–1.33)**
Sexual minority (Ref: No)	1.28 (0.77–2.14)	1.27 (0.85–1.91)	0.84 (0.44–1.60)	1.08 (0.96–1.21)
Married (Ref: No)	1.05 (0.62–1.76)	1.26 (0.81–1.96)	**0.18 (0.07–0.48)**	**1.15 (1.02–1.30)**
Having child (Ref: No)	1.29 (0.58–2.88)	1.31 (0.71–2.43)	**5.47 (1.43–20.96)**	1.02 (0.86–1.20)
Other tobacco use, past 6 months				
Cigarettes (Ref: No)	**9.09 (5.23–15.82)**	**5.19 (3.22–8.36)**	**5.46 (2.59–11.51)**	1.08 (0.96–1.20)
Other tobacco products (Ref: No) ^	**4.56 (2.59–8.04)**	**2.67 (1.61–4.45)**	**2.59 (1.07–6.23)**	0.98 (0.87–1.10)

^ Other tobacco included little cigars/cigarillos, hookah, and smokeless tobacco; boldface indicates *p* < 0.05. Never users were participants who reported no lifetime use; Former users were those who reported lifetime e-cigarette use but no use in the past 6 months; Recent users were those who reported past 6-month use but no past 30-day use; Current users were those who reported any past 30-day e-cigarette use. The term “Ref” denotes reference.

## Data Availability

Requests to access these datasets should be directed to the corresponding author. Data are not publicly available.

## Supplementary Materials

The following supporting information can be downloaded at: https://www.mdpi.com/article/10.3390/ijerph21020123/s1, Figure S1: Flowchart for participants included in final analysis by e-cigarette using status; Table S1: Baseline exposure to e-cigarette ads or reviews in the past 30 days and demographics by baseline e-cigarette use status; Table S2: Prevalence of e-cigarette use at follow-up waves by baseline e-cigarette use status and exposure to e-cigarette ads or reviews in the past 30 days; Table S3: Sensitivity analysis—adjusted associations between exposure to e-cigarette ads or reviews in the past 30 days and e-cigarette use at follow-up waves by baseline e-cigarette use status.
