# Supplementary material for: Exposure to E-Cigarette Advertisements or Reviews and E-Cigarette Use Progression: A Longitudinal Examination of Short-Term and Long-Term Associations among US Young Adults
Source: Int J Environ Res Public Health. 2024 Jan 23;21(2):123. doi: 10.3390/ijerph21020123 (PMC10887947; doi:10.3390/ijerph21020123)
Supplement: Supplementary file 1 [file ijerph-21-00123-s001.zip › ijerph-2747285-supplementary.pdf]

Supplementary Figure S1. Flowchart for participants included in final analysis by e-cigarette using status.

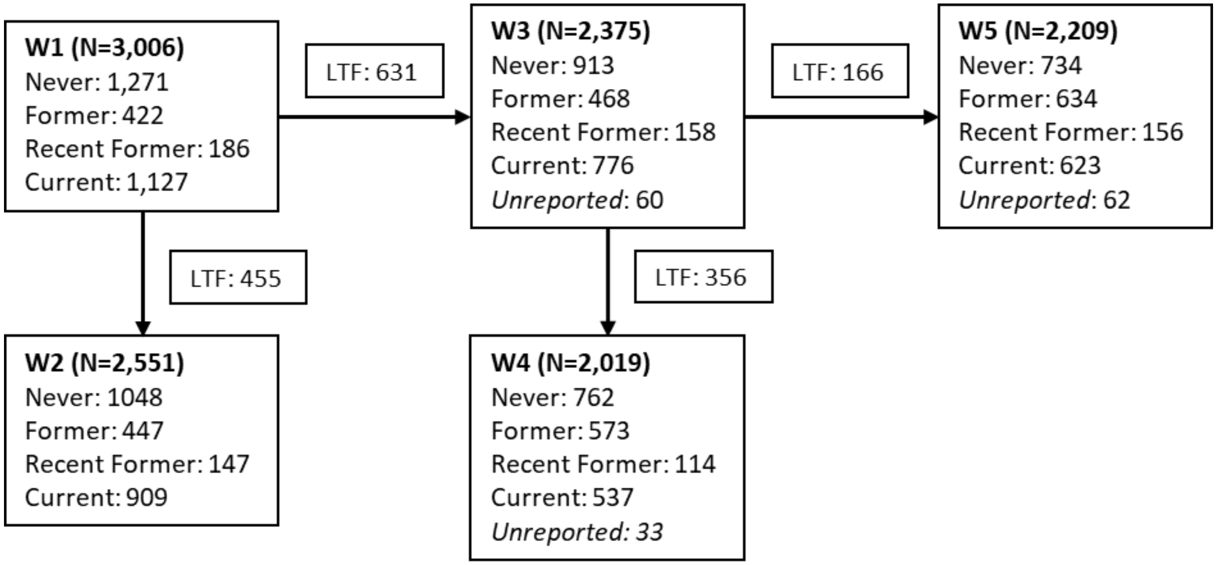

Note: LTF: loss to follow-up; Vertical arrows are 6-month/short-term follow-ups, and horizontal arrows are 12-month/long-term follow-ups.

Supplementary Table S1. Baseline (Wave 1 or Wave 3) exposure to e-cigarette ads or reviews in the past 30 days and demographics by baseline e-cigarette use status.

	Wave 1				Wave 3			
**Baseline characteristics**	**Never users N=1,271 % (n)**	**Former users N=422 % (n)**	**Recent former N=186 % (n)**	**Current users N=1,127 % (n)**	**Never users N=913 % (n)**	**Former users N=468 % (n)**	**Recent former N=158 % (n)**	**Current users N=776 % (n)**
Exposure to e-cigarette ads, N (%)								
Frequent	31.9 (405)	28.9 (122)	33.3 (62)	37.4 (422)	40.5 (370)	43.4 (203)	35.4 (56)	39.3 (305)
Not frequent	68.1 (866)	71.1 (300)	66.7 (124)	62.6 (705)	59.5 (543)	56.6 (265)	64.6 (102)	60.7 (471)
Exposure to e-cigarette reviews, N (%)								
Frequent	10.0 (127)	8.3 (35)	13.4 (25)	29.6 (333)	11.5 (105)	16.7 (78)	13.3 (21)	22.3 (173)
Not frequent	90.0 (1,144)	91.7 (387)	86.6 (161)	70.5 (794)	88.5 (808)	83.3 (390)	86.7 (137)	77.7 (603)
Age, M (SD)*	24.81 (4.65)	26.11 (4.55)	23.22 (4.02)	23.90 (4.78)	26.01 (4.66)	26.15 (4.56)	25.8 (4.47)	25.2 (4.76)
MSA, N (%)								
Atlanta	24.0 (305)	19.0 (80)	17.7 (33)	15.5 (175)	24.6 (225)	16.9 (79)	15.2 (24)	18.3 (142)
Boston	22.5 (286)	16.1 (68)	19.4 (36)	16.4 (185)	21.6 (197)	19.4 (91)	21.5 (34)	16.5 (128)
Minneapolis	15.8 (201)	19.0 (80)	25.3 (47)	19.1 (215)	14.8 (135)	19.2 (90)	22.2 (35)	17.7 (137)
Oklahoma City	9.9 (126)	11.1 (47)	6.5 (12)	11.9 (134)	9.3 (85)	10.0 (47)	9.5 (15)	9.8 (76)
San Diego	15.5 (197)	17.5 (74)	17.2 (32)	15.2 (171)	13.7 (125)	15.2 (71)	14.6 (23)	16.6 (129)
Seattle	12.3 (156)	17.3 (73)	14.0 (26)	21.9 (247)	11.5 (105)	14.7 (69)	11.4 (18)	17.9 (139)
Other	--	--	--	--	4.5 (41)	4.5 (21)	5.7 (9)	3.2 (25)
Sex, N (%)								
Male	39.8 (506)	40.1 (169)	33.9 (63)	47.3 (533)	37.4 (341)	38.7 (181)	42.4 (67)	46.1 (358)
Female	57.6 (732)	57.4 (242)	62.9 (117)	49.4 (557)	60.2 (550)	58.6 (274)	54.4 (86)	51.6 (400)
Other	2.6 (33)	2.6 (11)	3.2 (6)	3.3 (37)	2.4 (22)	2.8 (13)	3.2 (5)	2.3 (18)
Race, N (%)								
Non-Hispanic White	61.8 (761)	70.9 (292)	72.0 (131)	66.5 (733)	64.9 (576)	66.1 (300)	71.2 (109)	65.8 (500)
Non-Hispanic Black	6.3 (77)	5.8 (24)	3.9 (7)	3.5 (38)	5.9 (52)	5.5 (25)	3.3 (5)	4.0 (30)
Non-Hispanic Other	23.1 (285)	14.1 (58)	13.2 (24)	16.9 (186)	21.6 (192)	17.0 (77)	14.4 (22)	17.5 (133)
Hispanic	8.9 (109)	9.2 (38)	11.0 (20)	13.2 (146)	7.7 (68)	11.5 (52)	11.1 (17)	12.8 (97)
Education > College degree, N (%)	81.4 (1,022)	78.9 (329)	71.2 (131)	63.5 (709)	84.6 (762)	80.2 (372)	73.7 (115)	65.1 (499)
Employment, N (%)	62.0 (770)	69.9 (286)	63.1 (113)	66.1 (722)	61.5 (547)	67.4 (308)	55.1 (86)	67.3 (507)
Sexual minority, N (%)	24.3 (303)	34.3 (143)	38.8 (71)	34.9 (390)	23.2 (207)	33.4 (156)	41.1 (65)	33.5 (255)
Married, N (%)	34.4 (437)	42.7 (180)	25.3 (47)	36.9 (416)	35.9 (328)	41.5 (194)	32.3 (51)	37.1 (288)
Having child, N (%)	10.9 (139)	18.0 (76)	4.8 (9)	15.9 (179)	11.3 (103)	15.6 (73)	8.9 (14)	15.9 (123)
Other tobacco use, past 6 months, N (%)								
Cigarettes	7.7 (98)	32.7 (138)	42.5 (79)	60.2 (678)	4.4 (40)	27.4 (128)	41.8 (66)	56.3 (437)
Other tobacco products^	11.3 (143)	30.4 (128)	43.6 (81)	59.3 (666)	7.5 (68)	19.2 (90)	33.5 (53)	47.2 (366)

^ Other tobacco included little cigars/cigarillos, hookah, and smokeless tobacco. * M (SD) rather than % (n) reported.

Supplementary Table S2. Baseline covariates by exposure to e-cigarette ads or reviews in the past 30 days.

Baseline characteristics	Wave 1 exposure to e-cigarette ads		Wave 1 exposure to e-cigarette reviews		Wave 3 exposure to e-cigarette ads		Wave 3 exposure to e-cigarette reviews	
	Frequent N=1,011 % (n)	Not frequent N=1,995 % (n)	Frequent N=520 % (n)	Not frequent N=2,486 % (n)	Frequent N=953 % (n)	Not frequent N=1,422 % (n)	Frequent N=386 % (n)	Not frequent N=1,989 % (n)
Exposure to e-cigarette ads								
Frequent	100 (1,011)	0 (0)	75.2 (391)	24.9 (620)	100 (953)	0 (0)	81.1 (313)	32.2 (640)
Not frequent	0 (0)	100 (1,995)	24.8 (129)	75.1 (1,866)	0 (0)	100 (1,422)	18.9 (73)	67.8 (1,349)
Exposure to e-cigarette reviews								
Frequent	38.7 (391)	6.5 (129)	100 (520)	0 (0)	32.8 (313)	5.1 (73)	100 (386)	0 (0)
Not frequent	61.3 (620)	93.5 (1,866)	0 (0)	100 (2,486)	67.2 (640)	94.9 (1,349)	0 (0)	100 (1,989)
Age, M (SD)	24.57 (4.72)	24.55 (4.71)	24.60 (4.86)	24.55 (4.68)	24.61 (4.68)	24.70 (4.68)	25.14 (4.89)	24.57 (4.64)
MSA, N (%)								
Atlanta	17.7 (179)	20.8 (414)	14.0 (73)	20.9 (520)	20.8 (198)	21.3 (303)	20.5 (79)	21.2 (422)
Boston	19.4 (196)	19.0 (379)	17.5 (91)	19.5 (484)	19.1 (182)	21.6 (307)	18.7 (72)	21.0 (417)
Minneapolis	16.3 (165)	19.0 (378)	15.4 (80)	18.6 (463)	17.3 (165)	18.1 (257)	13.2 (51)	18.7 (371)
Oklahoma City	14.7 (149)	8.5 (170)	15.6 (81)	9.6 (238)	12.3 (117)	8.4 (120)	14.0 (54)	9.2 (183)
San Diego	15.6 (158)	15.8 (316)	17.3 (90)	15.5 (384)	15.0 (143)	16.4 (233)	17.1 (66)	15.6 (310)
Seattle	16.2 (164)	16.9 (338)	20.2 (105)	16.0 (397)	15.5 (148)	14.2 (202)	16.6 (64)	14.4 (286)
Sex, N (%)								
Male	44.7 (452)	41.1 (819)	47.3 (246)	41.2 (1,025)	41.7 (397)	41.0 (583)	43.5 (168)	40.8 (812)
Female	52.7 (533)	55.9 (1,115)	49.4 (257)	56.0 (1,391)	55.0 (524)	57.0 (811)	53.6 (207)	56.7 (1,128)
Other	2.6 (26)	3.1 (61)	3.3 (17)	2.8 (70)	3.4 (32)	2.0 (28)	2.9 (11)	2.5 (49)
Race, N (%)								
Non-Hispanic White	67.7 (677)	64.3 (1,250)	60.4 (305)	66.5 (1,612)	68.3 (636)	63.4 (877)	61.5 (232)	66.1 (1,281)
Non-Hispanic Black	4.2 (41)	5.4 (105)	4.8 (24)	5.0 (122)	3.8 (35)	5.7 (79)	3.7 (14)	5.2 (100)
Non-Hispanic Other	16.2 (160)	20.2 (393)	18.2 (92)	19.0 (461)	16.2 (151)	21.0 (290)	17.8 (67)	19.3 (374)
Hispanic	12.0 (118)	10.0 (195)	16.6 (84)	9.5 (229)	11.7 (109)	10.0 (138)	17.0 (64)	9.4 (183)
Education > College degree, N (%)	72.2 (725)	74.5 (1,466)	67.6 (349)	75.0 (1,842)	76.2 (719)	76.5 (1,074)	71.9 (276)	77.2 (1,517)
Employment, N (%)	67.2 (662)	63.4 (1,229)	68.8 (348)	63.8 (1,543)	65.1 (601)	63.0 (876)	68.9 (259)	62.9 (1,218)
Sexual minority, N (%)	31.1 (312)	30.4 (595)	29.9 (153)	30.8 (754)	32.0 (301)	28.9 (404)	32.1 (122)	29.8 (583)
Married, N (%)	40.4 (408)	33.7 (672)	43.1 (224)	34.4 (856)	40.4 (385)	34.7 (493)	43.0 (166)	35.8 (712)
Having child, N (%)	15.1 (153)	12.5 (250)	21.7 (113)	11.7 (290)	15.1 (144)	12.5 (177)	19.7 (76)	12.3 (245)
Other tobacco use, past 6 months, N (%)								
Cigarettes	37.0 (374)	31.0 (619)	52.1 (271)	29.0 (722)	29.5 (281)	30.3 (431)	39.6 (153)	28.1 (559)
Other tobacco products^	37.6 (379)	32.1 (639)	48.9 (252)	30.8 (766)	28.6 (272)	32.4 (460)	37.8 (145)	29.6 (587)

^ Other tobacco included little cigars/cigarillos, hookah, and smokeless tobacco.

Note: Significant Chi-Square/t-tests for bivariate associations ($p<.05$) for: 1) Wave 1 exposure to e-cigarette ads: Exposure to e-cigarette reviews, MSA, Race, Employment, Married, Having child, Past 6-month cigarette and other tobacco use; 2) Wave 1 exposure to e-cigarette reviews: all except for sexual minority; 3) Wave 3 exposure to e-cigarette ads: Exposure to e-cigarette reviews, MSA, Race, Married, Past 6-month other tobacco use; 4) Wave 3 exposure to e-cigarette reviews: all except for gender and sexual minority.

Supplementary Table S3. Sensitivity analysis - adjusted associations between exposure to e-cigarette ads or reviews in the past 30 days and e-cigarette use at follow-up waves by baseline e-cigarette use status.

	Short-term follow-up				Long-term follow-up			
**Baseline sample**	**Never users**	**Former users**	**Recent former users**	**Current users**	**Never users**	**Former users**	**Recent former users**	**Current users**
**E-cigarette use at follow-up waves**	**Initiated using**	**Became P6M user**	**Became current users**	**Number of days using**	**Initiated using**	**Became P6M user**	**Became current users**	**Number of days using**
	**aOR (95% CI)**	**aOR (95% CI)**	**aOR (95% CI)**	**IRR (95% CI)**	**aOR (95% CI)**	**aOR (95% CI)**	**aOR (95% CI)**	**IRR (95% CI)**
Exposure to e-cigarette ads	0.94 (0.73 - 1.21)	0.83 (0.69 - 1.01)	0.70 (0.48 - 1.02)	**0.95 (0.91 - 1.00)**	0.94 (0.75 - 1.18)	0.89 (0.76 - 1.06)	0.83 (0.58 - 1.19)	0.99 (0.94 - 1.05)
Exposure to e-cigarette reviews	1.11 (0.80 - 1.54)	1.16 (0.90 - 1.49)	**1.76 (1.13 - 2.73)**	**1.09 (1.04 - 1.14)**	1.09 (0.80 - 1.48)	0.99 (0.80 - 1.23)	1.24 (0.83 - 1.86)	**1.07 (1.01 - 1.13)**
Age	0.95 (0.90 - 1.01)	**0.93 (0.87 - 0.98)**	**0.89 (0.80 - 0.99)**	1.01 (1.00 - 1.02)	**0.91 (0.86 - 0.97)**	**0.90 (0.85 - 0.96)**	0.97 (0.88 - 1.07)	1.01 (0.99 - 1.02)
MSA								
Atlanta	Reference	Reference	Reference	Reference	Reference	Reference	Reference	Reference
Boston	1.03 (0.53 - 1.98)	**3.24 (1.50 - 7.02)**	2.45 (0.82 - 7.33)	**0.83 (0.69 - 0.99)**	0.85 (0.46 - 1.58)	1.83 (0.95 - 3.54)	1.98 (0.66 - 5.96)	**0.68 (0.56 - 0.83)**
Minneapolis	0.77 (0.34 - 1.76)	**3.21 (1.46 - 7.07)**	1.64 (0.53 - 5.03)	**1.24 (1.06 - 1.44)**	0.47 (0.20 - 1.14)	0.95 (0.46 - 1.95)	**3.63 (1.14 - 11.56)**	1.02 (0.86 - 1.20)
Oklahoma City	0.83 (0.33 - 2.03)	**4.80 (1.87 - 12.30)**	1.90 (0.37 - 9.78)	1.09 (0.90 - 1.31)	0.69 (0.28 - 1.71)	**2.73 (1.28 - 5.82)**	2.16 (0.42 - 10.99)	0.89 (0.72 - 1.09)
San Diego	1.27 (0.64 - 2.54)	**3.60 (1.58 - 8.22)**	2.89 (0.85 - 9.82)	1.03 (0.87 - 1.22)	1.22 (0.64 - 2.33)	1.55 (0.77 - 3.11)	3.14 (0.89 - 11.07)	0.92 (0.77 - 1.10)
Seattle	0.66 (0.26 - 1.66)	**2.69 (1.12 - 6.43)**	1.10 (0.29 - 4.09)	1.06 (0.90 - 1.25)	1.19 (0.56 - 2.51)	1.30 (0.63 - 2.68)	1.79 (0.42 - 7.69)	0.90 (0.75 - 1.08)
Other	--	0.98 (0.17 - 5.74)	--	0.65 (0.35 - 1.20)	0.41 (0.04 - 4.71)	0.52 (0.09 - 2.81)	13.15 (0.96 - 180.28)	0.74 (0.43 - 1.27)
Sex								
Male	1.01 (0.63 - 1.60)	0.74 (0.49 - 1.11)	0.88 (0.45 - 1.69)	0.93 (0.84 - 1.03)	0.92 (0.60 - 1.42)	0.95 (0.62 - 1.47)	0.98 (0.50 - 1.90)	**0.89 (0.80 - 1.00)**
Female	0.88 (0.18 - 4.37)	1.18 (0.43 - 3.18)	1.30 (0.21 - 7.85)	1.00 (0.73 - 1.37)	0.51 (0.09 - 2.93)	0.77 (0.25 - 2.38)	--	0.97 (0.67 - 1.41)
Other	Reference	Reference	Reference	Reference	Reference	Reference	Reference	Reference
Race								
Non-Hispanic White	Reference	Reference	Reference	Reference	Reference	Reference	Reference	Reference
Non-Hispanic Black	0.93 (0.33 - 2.62)	0.99 (0.36 - 2.71)	**7.86 (1.61 - 38.35)**	**0.37 (0.24 - 0.57)**	0.71 (0.26 - 1.97)	0.74 (0.29 - 1.89)	2.05 (0.28 - 14.93)	**0.37 (0.25 - 0.53)**
Non-Hispanic Other	1.37 (0.77 - 2.43)	1.10 (0.64 - 1.89)	1.22 (0.49 - 3.02)	**0.83 (0.72 - 0.95)**	1.29 (0.78 - 2.12)	0.97 (0.56 - 1.67)	1.51 (0.60 - 3.77)	**0.79 (0.67 - 0.93)**
Hispanic	1.44 (0.68 - 3.06)	0.59 (0.30 - 1.16)	0.78 (0.25 - 2.43)	**0.79 (0.68 - 0.92)**	0.92 (0.43 - 1.93)	0.79 (0.43 - 1.46)	0.86 (0.21 - 3.58)	**0.74 (0.62 - 0.88)**
Education (Ref: < college)	0.83 (0.47 - 1.48)	1.18 (0.68 - 2.05)	0.91 (0.39 - 2.13)	**0.84 (0.76 - 0.93)**	0.59 (0.35 - 1.02)	0.87 (0.53 - 1.43)	0.70 (0.31 - 1.57)	**0.85 (0.75 - 0.95)**
Employment (Ref: unemployed)	1.51 (0.89 - 2.54)	1.09 (0.67 - 1.77)	0.58 (0.29 - 1.15)	1.06 (0.95 - 1.18)	**1.86 (1.08 - 3.19)**	0.84 (0.54 - 1.31)	0.97 (0.47 - 2.01)	**1.16 (1.03 - 1.32)**
Sexual minority (Ref: No)	**2.02 (1.23 - 3.32)**	1.26 (0.78 - 2.02)	0.61 (0.32 - 1.17)	1.02 (0.91 - 1.13)	1.29 (0.77 - 2.14)	1.31 (0.88 - 1.96)	0.86 (0.45 - 1.65)	1.07 (0.96 - 1.21)
Married (Ref: No)	0.78 (0.45 - 1.35)	0.76 (0.48 - 1.21)	1.17 (0.47 - 2.87)	**1.16 (1.05 - 1.29)**	1.05 (0.62 - 1.76)	1.27 (0.82 - 1.97)	**0.18 (0.07 - 0.47)**	**1.16 (1.03 - 1.30)**
Having child (Ref: No)	0.85 (0.34 - 2.14)	1.75 (0.95 - 3.23)	2.46 (0.70 - 8.60)	0.98 (0.84 - 1.13)	1.28 (0.58 - 2.85)	1.36 (0.73 - 2.53)	**5.57 (1.48 - 20.98)**	1.01 (0.86 - 1.19)
Other tobacco use, past 6 months								
Cigarettes (Ref: No)	**10.99 (6.15 - 19.65)**	**4.54 (2.88 - 7.17)**	**4.03 (1.90 - 8.55)**	1.06 (0.96 - 1.17)	**9.01 (5.16 - 15.73)**	**5.21 (3.23 - 8.40)**	**5.54 (2.64 - 11.63)**	1.07 (0.96 - 1.20)
Other tobacco products (Ref: No)^	**3.64 (2.02 - 6.58)**	**3.92 (2.47 - 6.23)**	1.60 (0.75 - 3.41)	1.02 (0.92 - 1.12)	**4.55 (2.60 - 7.99)**	**2.78 (1.67 - 4.62)**	**2.65 (1.11 - 6.35)**	0.98 (0.88 - 1.10)

Notes: P6M=past 6-month. ^ Other tobacco included little cigars/cigarillos, hookah, and smokeless tobacco. Boldface indicates p<.05.
